# Supplementary material for: The interactome of intact mitochondria by cross-linking mass spectrometry provides evidence for coexisting respiratory supercomplexes
Source: Mol Cell Proteomics. 2017 Dec 8;17(2):216–32. doi: 10.1074/mcp.RA117.000470 (PMC5795388; doi:10.1074/mcp.RA117.000470)
Supplement: Supplemental Data [file supp_RA117.000470_133922_0_supp_23978_fzffwf.zip › spectra_annotation/mito_DR_spectra_annotation/343-1-12-1-39-1.pdf]

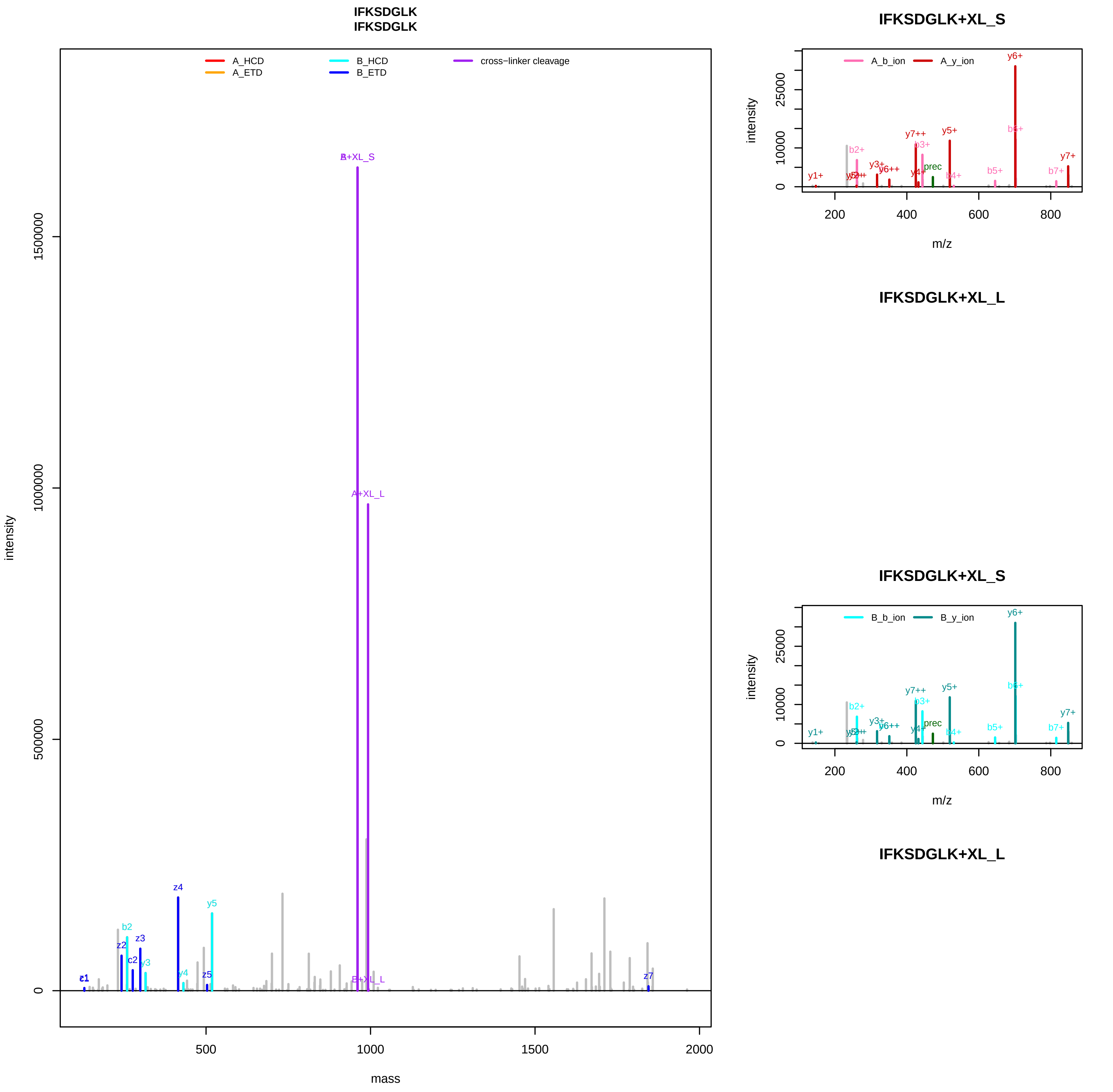
IFKSDGLK
IFKSDGLK
A_HCD
A_ETD
B_HCD
B_ETD
cross−linker cleavage
B+XL_S
A+XL_L
B+XL_L
c1
z1
z2
c2
b2
z3
y3
z4
y4
z5
y5
z7
intensity
mass
0
500000
1000000
1500000
500
1000
1500
2000
IFKSDGLK+XL_S
A_b_ion
A_y_ion
y1+
y2+
y3+
y6++
y7++
b3+
y4+
prec
y5+
b4+
b5+
b6+
y6+
b7+
y7+
b2+
intensity
m/z
0
10000
25000
200
400
600
800
IFKSDGLK+XL_L
IFKSDGLK+XL_S
B_b_ion
B_y_ion
IFKSDGLK+XL_L
